# Associations of cord blood biomarkers with infant colic and excessive crying

**DOI:** 10.3389/fped.2026.1767660

**Published:** 2026-04-10

**Authors:** Elisabeth M. Simonin, Karen M. Switkowski, Sheryl L. Rifas-Shiman, Kari C. Nadeau, Emily Oken, Jenifer R. Lightdale

**Affiliations:** 1 Department of Environmental Health, Harvard T. H. Chan School of Public Health, Boston, MA, United States; 2 Department of Biology, School of Sciences and Health Professions, Simmons University, Boston, MA, United States; 3 Division of Chronic Disease Research Across the Lifecourse, Department of Population Medicine, Harvard Medical School and Harvard Pilgrim Health Care Institute, Boston, MA, United States; 4 Division of Gastroenterology, Hepatology, and Nutrition, Department of Pediatrics, Stanford School of Medicine, Stanford University, Palo Alto, CA, United States; 5 Division of Gastroenterology, Hepatology and Nutrition, Department of Pediatrics, Boston Children's Hospital, Boston, MA, United States; 6 Department of Pediatrics, Harvard Medical School, Boston, MA, United States

**Keywords:** biomarker, cord blood microbiome, *Gammaproteobacteria*, infant colic, trans fatty acids

## Abstract

**Introduction:**

There are currently no known biomarkers associated with the diagnosis of infant colic, a common early disorder of gut brain interaction (DGBI) that has been found to predict adverse health outcomes, including atopy, migraines and other DGBIs. Infant colic manifests as unsoothable crying and is perceived to be associated with abdominal pain, differentiating it from other crying behaviors. Prior studies have postulated it may involve microbial dysbiosis as well as immunological and neurological dysregulation. The aim of our study was to investigate the associations of cord blood biomarkers at birth with parent reports of colic and excessive crying behaviors at 6 months of age.

**Methods:**

We used available data from Project Viva a pre-birth cohort based in the greater Boston, MA area. All infants were born between 1999 and 2002.

**Results:**

Among participants with information on infant colic and cord blood biomarkers (*n* = 405), we found higher trans fatty acids and an increased abundance of *Gammaproteobacteria* signature in cord blood from infants with colic and those with excessive crying without colic, compared to those unaffected by colic. The majority of inflammatory and immune system cord blood biomarkers previously measured, including metabolites and cytokine stimulation, showed no association with either colic or excessive crying.

**Discussion:**

This exploratory study examined cord blood biomarkers of inflammation or immune dysregulation to support the underlying mechanism of infant colic, and identified trans fatty acid levels and *Gammaproteobacteria* microbial signatures as possible candidate predictors. On the other hand, we also found a lack of association with most of the cord blood immune and neurological biomarkers that we assessed. In turn, we propose that colic biomarkers may be present closer to its manifestation as a clinical condition of early infancy.

## 1 Introduction

Infant colic is a common clinical condition in newborns that has been defined as an early disorder of gut-brain interaction (DGBI) (1). Colic manifests with unsoothable crying and signs of abdominal distress. Colic typically emerges at 2–3 weeks of age, peaks during the first 3 months of life, resolves by 6 months, and has historically been considered a benign and self-limited condition (1–3). However, recent research has demonstrated that colic may be an early predictor of adverse health outcomes later in childhood and adolescence, particularly atopic conditions, migraines, and functional gastrointestinal disorders (2–4). To date, underlying mechanisms that could link colic in infancy with later health outcomes remain unknown. In this study, we hypothesized that cord blood biomarkers of neurological and immune system dysfunction, as well as microbial signatures, could be associated with the pathogenesis of infant colic.

We considered our study to be exploratory and extensively reviewed the literature to identify candidate biomarkers for analysis. We found a number of studies that have suggested that colic may be associated with inflammatory conditions, immune dysregulation, and neurodivergence (3, 5, 6). More specifically, these studies suggest that a history of colic may be associated with increased likelihood of childhood and adolescent functional gastrointestinal and atopic disorders (7, 8), recurrent abdominal pain (9), migraine headaches (10), attention deficit hyperactivity disorder (11), allergic rhinitis, asthma, pollinosis, atopic eczema and food allergy (9). In addition, parental factors such as maternal stress (12, 13) and familial allergic diseases (2), may be associated with colic.

Our group has previously described epidemiological risk factors for infant colic that are distinct from risk factors associated with excessive crying without apparent abdominal distress (6, 14). Specifically, we have found that maternal atopy, postpartum depression, and persistent nausea are risk factors for the development of colic (14). Additionally, being first born, low birthweight, and preterm are risk factors for both colic and excessive crying without colic (14). These findings have strengthened our belief that colic and excessive crying are distinct conditions.

We hypothesized that our findings of different prenatal predictors for colic suggest the possibility of biological alterations *in utero* that predispose a fetus to develop one or another in infancy. Specifically, we considered a set of candidate cord blood biomarkers that are involved in inflammatory and behavioral pathways, as well as microbiome and fatty acid biomarkers, which could indicate dysbiosis. To test our hypothesis, we used data on maternal pre- and post-natal factors, biomarkers in cord blood samples collected at delivery from mothers in the Project Viva cohort, and data on colic and crying behavior in their infants, which mothers reported at 6 months of age (15). Our primary aim was to assess if any of our candidate cord blood biomarkers were associated with maternal reports of colic or with excessive crying compared with neither condition in the first six months of life, with the ultimate goal to advance our current understanding of the underlying mechanisms of infant colic and excessive crying.

## 2 Methods

### 2.1 Study population

We studied a subset of infants enrolled in Project Viva, a longitudinal pre-birth cohort based in the greater Boston region of Eastern Massachusetts. Briefly, pregnant women were enrolled during early pregnancy between 1999 and 2002 from eight obstetric offices of a multispecialty group practice in eastern Massachusetts. Detailed recruitment and exclusion criteria have been previously described (15). Research assistants conducted in-person research visits with the mothers during pregnancy, in the hospital after delivery, and at approximately six months after the birth of the child. Mothers provided written informed consent for themselves and on behalf of their infant. The Harvard Pilgrim Health Care Institutional Review Board approved all procedures (15).

### 2.2 Assessment of excessive crying and infant colic

We assessed excessive crying and infant colic with questions about the maternal perception of crying behavior and abdominal discomfort, respectively. This has been previously described (14). Excessive crying was assessed by three factors: crying frequency, ease of calming, and problematic crying. Infants with at least two of these three factors were considered to have “excessive crying”. Colic was evaluated by questions about the maternal perception of abdominal discomfort. Specifically, mothers were asked “Has your baby ever had times when he/she appears to be in agony, screams, draws his/her legs up to his/her body, and can't be calmed?”. If answered “yes”, then mothers were asked a follow-up question about frequency of this occurrence. We considered infants whose mothers reported that this occurred “sometimes” or “often” to have colic.

Our previous work (14) categorized infants into three groups: colic, excessive crying, and unaffected. For this study, we categorized the infants into four groups: 1) unaffected with no excessive crying or colic (“Unaffected”), 2) excessive crying with no signs of colic (“Excessive crying only”), 3) colic without excessive crying (“Colic only”), and 4) colic with excessive crying (“Colic and crying”) after preliminary results suggested differences in cord blood biomarker results in the colic groups with and without excessive crying. Sample sizes varied across biomarker assays, which were completed in different analytic laboratories at different times and reported in detail in prior publications. We have summarized prior Viva work on biomarkers in Supplementary Table 1 and have provided the sample size of each group for each biomarker analysis below.

### 2.3 Cord blood collection and cell isolation

For non-emergency deliveries in Project Viva that occurred at one of two study hospitals, the delivering clinician collected umbilical cord whole blood by needle/syringe from the umbilical vein into EDTA tubes. Blood samples were stored at 4 °C and processed within 24 h (16, 17). Cord blood mononuclear cells (CBMC) were isolated by density gradient centrifugation with Ficoll-Hypaque Plus (Pharmacia, Uppsala, Sweden). After centrifugation, plasma was removed and stored in liquid nitrogen until further processing. CBMC were washed and resuspended in RPMI-1640 10% human serum (Biowhittaker, Walkersville, MD), and frozen in liquid nitrogen until further processing. For the purposes of the present paper, we used only extant data on analyses previously conducted. We briefly summarize methods below and all have been previously reported in other publications.

### 2.4 Measurement of lymphocyte proliferation and cytokine production

To measure lymphocyte proliferation and cytokine secretion, CBMC were cultured in the presence of different stimuli and subsequently analyzed (18). CBMC were cultured at 0.5 × 10^6 cells/well for 72 h in the presence of 30 µg/mL cockroach allergen (Bla g 2), 30 µg/mL house dust mite allergen (Der f 1; Indoor Biotechnologies, Charlottesville, VA), 100 µg/mL ovalbumin allergen (OVA), 5 µg/mL PHA mitogen (Sigma Aldrich, St. Louis, MO), or media control. Proliferation was measured by tritiated thymidine (^3^H-TdR) and the mean counts per minute values for each condition was calculated. A stimulation index (SI) was calculated as the ratio of the mean counts per minute of stimulated lymphocytes divided by the mean counts per minute of unstimulated lymphocytes. Lymphocyte proliferation was measured in samples from unaffected (*n* = 183), excessive crying only (*n* = 29), colic only (*n* = 45) and colic and crying (*n* = 25) groups.

Supernatants were also collected at 72 h and secreted IL-6, IL-10, IL-13, IFN-*γ*, and TNF*α* were measured by ELISA (Endogen, Rockford, IL). Cytokine production was media corrected before analysis. In addition, we grouped the cytokine data into groups “undetectable”, “< median detectable level”, and “> median detectable level” for IL-6, IL-10 and TNF*α* and we dichotomized the data into “undetectable” and “detectable” for IL-13 and IFN-*γ*. We repeated the analysis on the cytokine data for both the media corrected numeric values and the 2- or 3-category variables. Both analyses produced similar results and we report the statistics generated from the numeric variables, incremented per interquartile range (IQR). OVA-stimulation was excluded from the cytokine measurements in this analysis (18). Cytokine production was measured in samples from unaffected (*n* = 147), excessive crying only (*n* = 24), colic only (*n* = 35) and colic and crying (*n* = 19) groups.

### 2.5 Measurement of blood fatty acids and metabolites

To measure fatty acids (FA), cord blood plasma was analyzed by gas-liquid chromatography (18). Injected standards (NuCheck Prep, Elysium, MN) were used to identify and quantify peak retention times and area percentages. Specific FA levels were calculated using ChemStation software version A.08.03 (Agilent, Santa Clara, CA) and are reported as percentage of total FA. FA analysis was performed for each individual FA measured, and for grouped omega-3, omega-6 and trans FA. “Omega-3 FA” included docosahexaenoic acid (DHA), monounsaturated fatty acid 9c-octadecenoic acid (oleic acid, M6), saturated fatty acids hexadecenoic acid (palmitic acid, S4) and octadecanoic acid (stearic acid, S6), 5c,8c,11c,14c17c-eicosapentaenoic acid (EPA). “Omega-6 FA” included arachidonic acid (AA) and linoleic acid (LA). Saturated fatty acids docosanoic acid (S10), and tricosanoic acid (S11), and monounsaturated fatty acids 9c-hexadecenoic acid (palmitoleic acid, M3), 11c-octadecenoic acid (M7), and 15c-tetrasenoic acid (nervonic acid, M12) were also included. FA were measured in samples from unaffected (*n* = 170), excessive crying only (*n* = 23), colic only (*n* = 43) and colic and crying (*n* = 25) groups.

To measure metabolites, cord blood plasma was analyzed by untargeted metabolomic profiling using multi-platform mass spectroscopy (Metabolon, Durham, NC) (16, 19). Metabolites (*n* = 415 metabolites) were measured, log2-transformed and reported as concentrations in arbitrary units. We imputed missing values with ½ the minimum detected value for each metabolite. Metabolites that were undetectable in >50% of samples were removed from the analysis (*n* = 27 metabolites). Values were log2-transformed for analysis. Principal components analysis (PCA) was used for dimension reduction, and we used standard criteria of the Scree plot “break” and Eigenvalues >1 to determine the number of factors to retain (19). Metabolites were measured in samples from unaffected (*n* = 60), excessive crying only (*n* = 11), colic only (*n* = 11) and colic and crying (*n* = 8) groups.

### 2.6 DNA isolation, 16S rDNA amplicon sequencing and analysis

Bacterial-derived DNA in cord blood samples was isolated, amplified and quantified (20, 21). Briefly, DNA was extracted from 1 mL cord blood serum using a QIAmp DNA Blood Midi Kit (Qiagen, Hilden, Germany). Molecular-grade water was run in parallel as a negative control. DNA concentration and quality were measured by Qubit dsDNA High Sensitivity Dye (Invitrogen, Carlsbad, CA) on a 2100 Bioanalyzer (Agilent, Santa Clara, CA).

V1-V3 regions of the rRNA gene were amplified and barcoded with the NEXTflex™ 16s V1-V3 Amplicon-Seq Kit (BIOO Scientific, Austin, TX) to generate 16s ribosomal DNA (rDNA) libraries. As expected, the negative control did not yield a library. Libraries were pooled and size selected by Pippin Prep™ 1.5% Agarose Dye-Free Pippin Gel Cassette (Sage Science, Beverly, MA) for amplicons between 550 and 700 base pairs (bp). Libraries were sequenced at 15pM with 5% PhiX on Illumina's MiSeq platform using the MiSeq Reagent v3-600 cycle kit (Illumina, San Diego, CA) with paired-end 301 read length. Microbial 16S sequencing was performed in samples from unaffected (*n* = 116), excessive crying only (*n* = 17), colic only (*n* = 27) and colic and crying (*n* = 15) groups.

### 2.7 16S rRNA microbial quantification, normalization and analysis

To quantify and normalize microbial diversity, and for operational taxonomic unit (OTU) annotation, rDNA sequencing data was processed by Quantitative Insights into Microbial Ecology (QIIME2) version 2024.5.0, using a procedure similar to one of our prior studies (22) and the QIIME2 recommended protocol (23). Briefly, low quality bases were trimmed, and taxonomy was assigned using the GreenGenes2 pre-trained naïve Bayes taxonomic classifier (gg_2022_10_backbone_full_length). Sample sequences with <10,000 nucleotides were removed.

Data were rarefied to an even sampling depth of 10,000 sequences, which retained all 175 samples and 22.87% of the total features. Alpha (*α*) Diversity was measured by Shannon and Faith indices, and Beta (*β*) Diversity metrics were calculated using permutational multivariate analysis of variance (PERMANOVA) with weighted UniFrac distance in the R package vegan (version 2.6-6.1) and function adonis2(). PERMANOVA was run using R Version 4.2.2.

### 2.8 Statistical analysis

To test associations between cord blood biomarkers and the 4-level colic/crying variable, we analyzed each biomarker separately. We examined associations of lymphocyte proliferation SI, secreted cytokine concentration, metabolite factor scores and FA percentages with colic/crying by unadjusted multinomial logistic regression. Further adjustment for maternal education, maternal history of atopy, and child race/ethnicity had a negligible influence on the odds ratios (95% CIs) and therefore unadjusted multinomial logistic regression was used. For each biomarker, we compared biomarker measurements among the four-category groups. The majority of our presented data compares the biomarker in each colic/crying group with the unaffected group as the reference group. As we were interested in exploring associations and not identifying causal relationships, we did not include covariates or adjust our analyses for potential confounders. We present the 95% confidence intervals (95% CI) and relative risk ratios (RR) for each colic/crying group compared to the unaffected group.

To calculate differentially abundant bacterial taxa between the 4-level colic variable, two methods were used: the ANCOM method (24) and the discrete FDR (DS-FDR) method (25). DS-FDR uses permutations to account for multiple testing and is designed to achieve higher statistical power in samples with sparse microbiomes (22). OTUs present in less than 4 samples were excluded from analysis.

Logistic regression was run using R version 4.2.2. Microbial diversity metrics, described above, and ANCOM were calculated using QIIME2 version 2024.5.0.

## 3 Results

**Figure 1:**
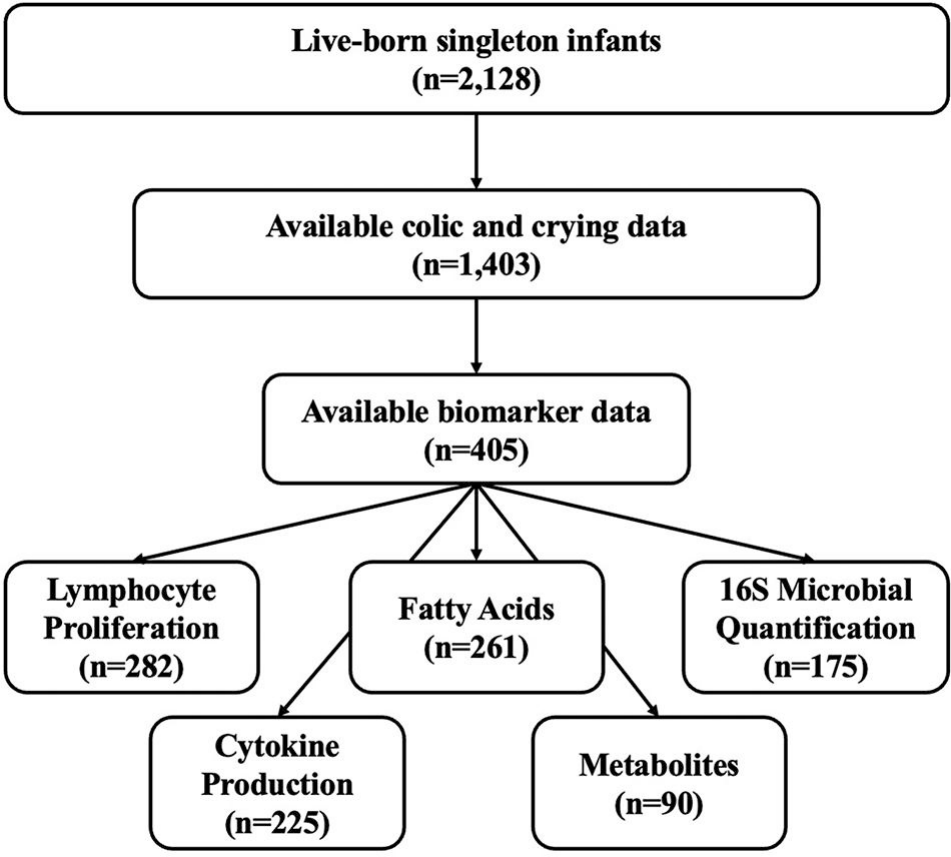
Participant flow from the project Viva cohort. Biomarkers were measured in a subset of participants in the Project Viva cohort. First, the cohort was filtered to only include participants with colic and crying data (*n* = 1,403). Of these, a subset had available biomarker data for at least one measured biomarker (*n* = 405).

Of the 2,128 live-born singleton infants in the Project Viva cohort, 1,403 infants had available data on crying behaviors and signs of colic during the six-month infancy visit. The demographics of each colic/crying group are reported in Table 1. Among the subset of infants with information on colic and crying behaviors and cord blood biomarkers, we found no association of sex, gestational age, delivery route or race and ethnicity with the four colic and crying categories. A subset of 405 infants (*n* = 264 unaffected, *n* = 41 with excessive crying only, *n* = 62 with colic only, *n* = 38 with colic and crying) had cord blood biomarker measurements for at least one of the biomarkers analyzed in this study. The majority of the infants (*n* = 302) had biomarker measurements for more than 1 biomarker. Within this subset of 405 infants, we analyzed biomarker data for possible associations with the colic and crying groups (Figure 1, Supplementary Table 1).

**Table 1:** Characteristics of 405 Project Viva infants.

Variables	Unaffected	Excessive crying only	Colic only	Colic and crying
Sample size^a^ (*n*)	264	41	62	38
Sex				
Female % (*n*)	48.9% (*n* = 129)	34.1% (*n* = 14)	46.8% (*n* = 29)	42.1% (*n* = 16)
Male % (*n*)	51.1% (*n* = 135)	65.9% (*n* = 27)	53.2% (*n* = 33)	57.9% (*n* = 22)
Gestational Age, weeks mean (SD, range)	39.7 (SD 1.39, range 31.3–42.1)	39.5 (SD 1.49, range 35.4–41.9)	39.7 (SD 1.69, range 30.9–41.7)	39.3 (SD 2.28, range 30.9–41.9)
Delivery Route				
Vaginal % (*n*)	81.2% (*n* = 214)	78.0% (*n* = 32)	83.9% (*n* = 52)	78.9% (*n* = 30)
Caesarean % (*n*)	18.0% (*n* = 48)	22.0% (*n* = 9)	16.1% (*n* = 10)	21.1% (*n* = 8)
Not Recorded % (*n*)	0.8% (*n* = 2)	0%	0%	0%
Race and Ethnicity^b^				
Non-Hispanic White % (*n*)	72.3% (*n* = 191)	65.9% (*n* = 27)	71.0% (*n* = 44)	65.8% (*n* = 25)
Not non-Hispanic White % (*n*)	27.6% (*n* = 73)	34.1% (*n* = 14)	29.0% (*n* = 18)	34.2% (*n* = 13)

^a^ This table includes all participants in the Project Viva cohort with information on colic and crying behaviors, and at least one biomarker measurement included in the analysis.

^b^ Race and ethnicity were assigned retrospectively based on the race and ethnicity reported by the child in adolescence. Missing race and ethnicity data in adolescence was supplemented with the mother's report of child race and ethnicity at 3 years. Race and ethnicity is dichotomized as “Non-Hispanic White” or “Not non-Hispanic White” because the rates of colic are highest in non-Hispanic White participants and similar among other groups (14). “Not non-Hispanic White” included “Black”, “Hispanic”, “Asian” and “>1 race or other”.

### 3.1 Fatty acids in cord blood compared between colic groups

**Figure 2:**
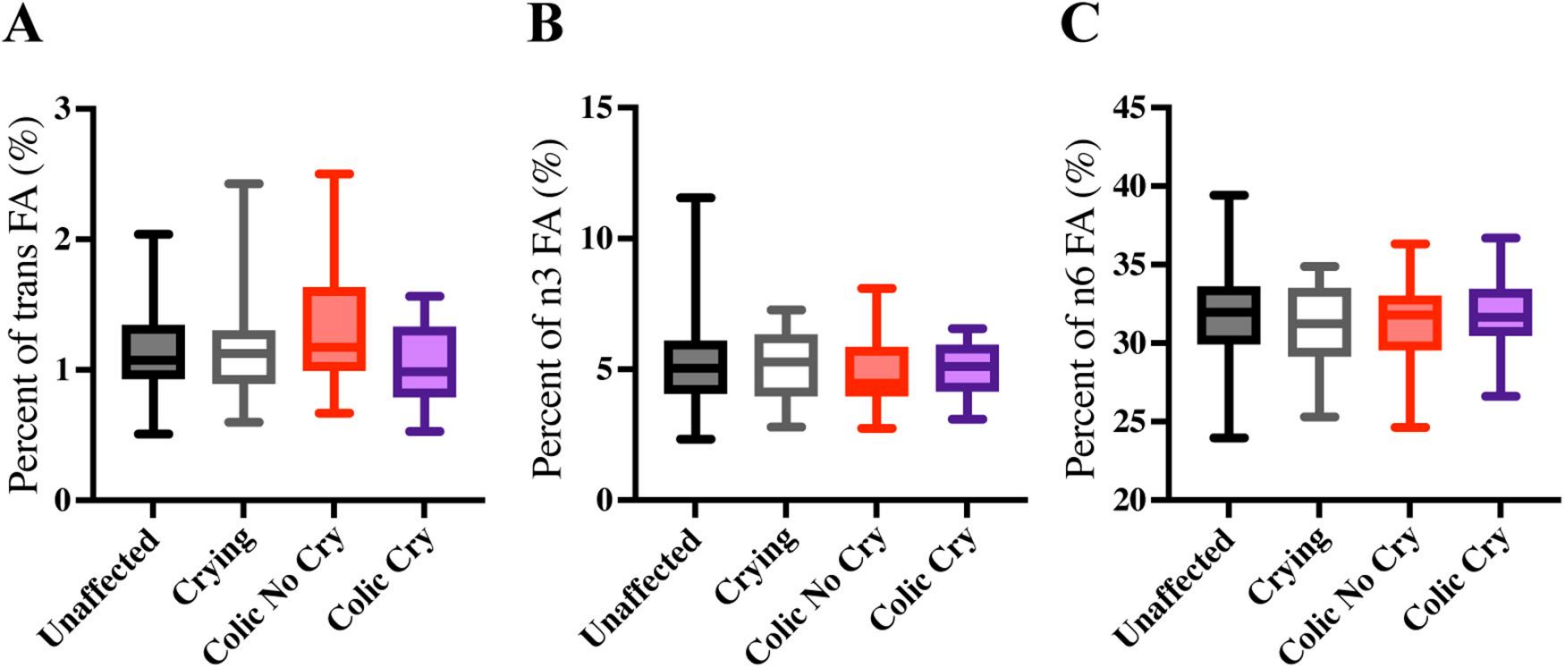
Cord blood trans fatty acid levels differ between colic groups. Fatty acid levels were measured and compared between unaffected (gray bars, *n* = 170), crying only (white bars, *n* = 23), colic only (red bars, *n* = 43), and colic and crying (purple bars (*n* = 25) groups. The percent out of total fatty acids measured for **(A)** trans fatty acids, **(B)** omega-3 (n3) fatty acids, and **(C)** omega-6 (n6) fatty acids are compared. Boxes plot 25% percentile, median and 75% percentile and whiskers plot minimum and maximum.

Fatty acid composition in cord blood differed between the colic only group and other colic/crying groups (Figure 2). First, we compared the percentage of fatty acids grouped by type (trans, omega-3 and omega-6) between colic/crying groups. Trans fatty acids were highest among the colic only group [mean 1.30% of total FA, SD 0.42, 95% CI (1.18, 1.43)] when compared to the unaffected group (mean 1.15% of total FA, SD 0.30, 95% CI [1.10, 1.20], and when compared to the colic and crying group [mean 1.06% of total FA, SD 0.29, 95% CI (0.94, 1.18)] (Figure 2A). The risk ratio (RR) for every 1% increase in cord blood trans fatty acids was 3.51 for the colic only group vs. the unaffected group and is 9.87 for the colic only group vs. the colic and crying group (Table 2). There was no difference in omega-3 or omega-6 fatty acids between colic/crying groups (Figures 2B,C, Table 2).

**Table 2:** Association of cord blood biomarkers with colic/crying groups compared to the unaffected group.

Category	Biomarker	Crying only	Colic only	Colic and Crying
		OR (95% CI)^b^^,^^c^		
Fatty Acids^a^	Trans fatty acids	0.86 (0.21, 3.46)	**3.51** **(1.37, 9.01)**	0.36 (0.08, 1.59)
	Omega-3 fatty acids	0.95 (0.68, 1.34)	0.81 (0.62, 1.08)	0.88 (0.63, 1.23)
	Omega-6 fatty acids	0.89 (0.75, 1.05)	0.92 (0.81, 1.05)	0.98 (0.83, 1.16)
	DHA	0.92 (0.61, 1.41)	0.82 (0.59, 1.15)	0.86 (0.57, 1.30)
	AA	0.84 (0.69, 1.03)	0.94 (0.81, 1.10)	0.93 (0.77, 1.14)
	EPA	0.73 (0.13, 4.03)	0.25 (0.06, 1.10)	0.88 (0.18, 4.38)
	LA	1.08 (0.82, 1.42)	0.93 (0.75, 1.16)	1.11 (0.85, 1.45)
	M12	0.49 (0.13, 1.94)	0.39 (0.13, 1.16)	1.20 (0.37, 3.86)
	M3	1.30 (0.81, 2.11)	1.10 (0.76, 1.58)	0.94 (0.59, 1.48)
	M6	1.11 (0.89, 1.39)	0.99 (0.82, 1.18)	1.09 (0.88, 1.36)
	M7	0.66 (0.21, 2.09)	0.77 (0.32, 1.84)	1.77 (0.61, 5.08)
	S10	0.38 (0.06, 2.19)	0.45 (0.12, 1.72)	1.29 (0.27, 6.19)
	S11	0.68 (0.08, 5.65)	0.54 (0.09, 3.17)	1.17 (0.23, 5.85)
	S4	1.09 (0.89, 1.32)	**1.18** (**1.01, 1.37)**	1.03 (0.85, 1.24)
	S6	1.04 (0.67, 1.62)	0.95 (0.68, 1.34)	0.99 (0.65, 1.51)
Metabolites	Factor 1	0.96 (0.50, 1.84)	1.26 (0.69, 2.30)	1.33 (0.68, 2.61)
	Factor 2	1.40 (0.77, 2.56)	**1.77** (**1.01, 3.12)**	1.06 (0.49, 2.31)
	Factor 3	1.09 (0.61, 1.94)	1.13 (0.63, 2.00)	1.23 (0.65, 2.33)
	Factor 4	1.00 (0.55, 1.84)	0.95 (0.52, 1.76)	0.86 (0.42, 1.76)
	Factor 5	0.72 (0.37, 1.40)	0.89 (0.49, 1.64)	1.02 (0.53, 1.97)
	Factor 6	0.59 (0.29, 1.21)	1.36 (0.66, 2.83)	0.52 (0.23, 1.15)
Bla g 2 stimulation	Lymphocyte Proliferation	1.11 (0.87, 1.41)	1.02 (0.80, 1.29)	0.87 (0.58, 1.29)
	IL-6 production	0.94 (0.61, 1.44)	0.85 (0.58, 1.26)	0.87 (0.53, 1.42)
	IL-10 production	0.91 (0.76, 1.09)	0.97 (0.85, 1.10)	1.04 (0.91, 1.17)
	IL-13 production	1.00 (0.98, 1.01)	0.98 (0.95, 1.00)	1.00 (0.99, 1.01)
	TNF-a production	0.68 (0.40, 1.13)	0.96 (0.64, 1.45)	1.06 (0.63, 1.79)
	IFN-*γ* production	0.96 (0.88, 1.05)	0.97 (0.93, 1.02)	1.00 (0.99, 1.01)
Der f 1 stimulation	Lymphocyte Proliferation	1.03 (0.86, 1.24)	0.98 (0.83, 1.16)	0.91 (0.70, 1.18)
	IL-6 production	1.01 (0.75, 1.37)	0.82 (0.55, 1.24)	0.69 (0.38, 1.26)
	IL-10 production	0.94 (0.80, 1.10)	0.94 (0.82, 1.08)	1.01 (0.87, 1.17)
	IL-13 production	1.00 (1.00, 1.01)	1.00 (0.99, 1.01)	0.98 (0.94, 1.02)
	TNF-a production	0.87 (0.50, 1.52)	0.95 (0.60, 1.51)	0.76 (0.39, 1.45)
	IFN-γ production	0.99 (0.97, 1.02)	0.90 (0.74, 1.09)	1.00 (0.98, 1.01)
OVA stimulation	Lymphocyte Proliferation	1.33 (0.87, 2.05)	1.07 (0.67, 1.70)	1.20 (0.71, 2.00)
PHA stimulation	Lymphocyte Proliferation	1.00 (1.00, 1.01)	1.00 (0.99, 1.01)	1.00 (1.00, 1.01)
	IL-6 production	1.03 (0.93, 1.15)	**0.53** (**0.29, 0.96)**	0.77 (0.42, 1.41)
	IL-10 production	0.85 (0.60, 1.19)	0.95 (0.75, 1.19)	1.16 (0.95, 1.42)
	IL-13 production	0.67 (0.31, 1.47)	1.01 (0.57, 1.81)	0.94 (0.43, 2.05)
	TNF-a production	0.96 (0.53, 1.73)	1.10 (0.67, 1.80)	1.41 (0.77, 2.61)
	IFN-γ production	0.98 (0.93, 1.03)	0.98 (0.94, 1.02)	1.02 (0.99, 1.05)

^a^ Fatty acid abbreviations are as follows: DHA, docosahexaenoic acid; AA, arachidonic acid; EPA, 5c,8c,11c,14c17c-eicosapentaenoic acid; LA, linoleic acid; M12, 15c-tetrasenoic acid (nervonic acid); M3, 9c-hexadecenoic acid (palmitoleic acid); M6, 9c-octadecenoic acid (oleic acid); M7, 11c-octadecenoic acid; S10, docosanoic acid; S11, tricosanoic acid; S4, hexadecenoic acid (palmitic acid); and S6, octadecanoic acid (stearic acid).

^b^ Odds Ratios (OR) and 95% Confidence Intervals (95% CI) for a one-unit change in each biomarker were calculated by exponentiating the coefficient for each colic/crying group compared to the unaffected group. Fatty acid units were reported as percentage of total fatty acids measured, metabolite units were reported as log2-transformed concentrations measured in arbitrary units, lymphocyte proliferation units were reported as the stimulation index (SI), and media-corrected cytokine concentration units were reported as pg/mL and analyzed per interquartile range (IQR).

^c^ Bolded OR (95% CI) have *p*-values <0.05.

We also compared specific fatty acids between colic/crying groups. First, we compared all FA with an abundance >1% of the total FA measured. These included omega-3 fatty acid docosahexaenoic acid (DHA), monounsaturated fatty acid 9c-octadecenoic acid (oleic acid, M6), saturated fatty acids hexadecenoic acid (palmitic acid, S4) and octadecanoic acid (stearic acid, S6), and omega-6 fatty acids arachidonic acid (AA) and linoleic acid (LA). There were no significant associations of any of these fatty acids, DHA, M6, S4, S6, AA or LA, with the colic/crying groups (Table 2).

The FA with an abundance <1% of the total FA included omega-3 fatty acid 5c,8c,11c,14c17c-eicosapentaenoic acid (EPA), saturated fatty acids docosanoic acid (S10), and tricosanoic acid (S11), monounsaturated fatty acids 9c-hexadecenoic acid (palmitoleic acid, M3), 11c-octadecenoic acid (M7), and 15c-tetrasenoic acid (nervonic acid, M12). There were no significant associations of any of these fatty acids, EPA, S10, S11, M3, M7 or M12, with the colic/crying groups (Table 2).

### 3.2 Microbial DNA signatures in cord blood compared between colic groups

**Figure 3:**
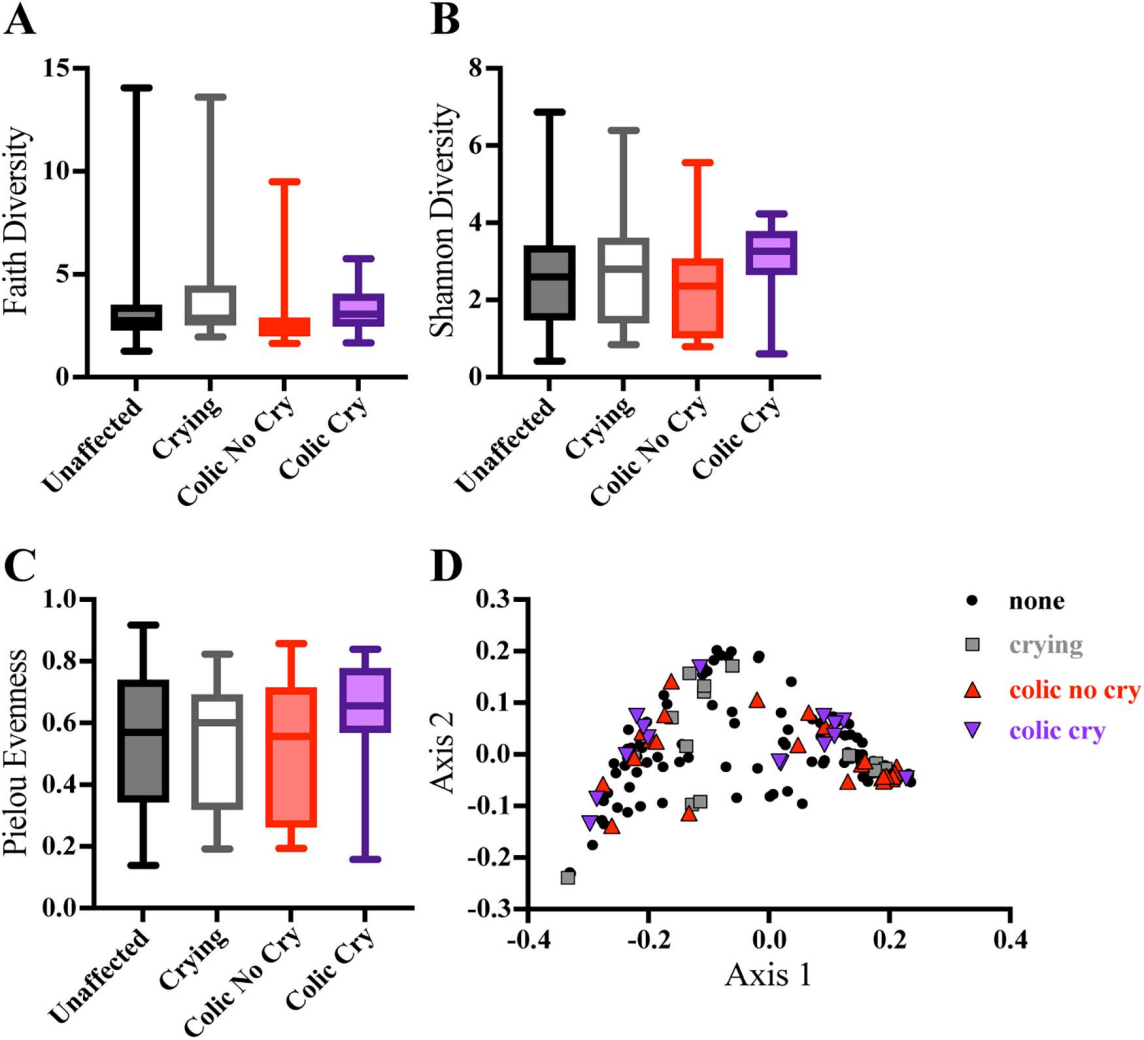
Cord blood microbiome alpha diversity is associated with colic groups. 16S sequencing alpha diversity metrics were compared using unpaired, nonparametric Mann–Whitney tests. Diversity metrics were compared between unaffected (gray bars and black circles, *n* = 116), crying only (white bars and gray squares, *n* = 17), colic only (red bars and red triangles, *n* = 27), and colic and crying (purple bars and purple inverse triangles (*n* = 15) groups. **(A)** Faith Diversity, **(B)** Shannon Diversity, and **(C)** Pielou Evenness. Beta diversity was compared by **(D)** UMAP using the weighted UniFrac metric and PERMANOVA. Boxes plot 25% percentile, median and 75% percentile and whiskers plot minimum and maximum.

Microbial DNA diversity metrics between groups suggested variability in their signature similarity and differences (Figure 3) (26, 27). The colic only group had lower Alpha diversity compared to the unaffected group (Faith diversity *p* = 0.011) and compared to the colic and crying group (Faith diversity *p* = 0.011, Shannon diversity *p* = 0.008) (Figures 3A,B). There was no difference in Pielou Evenness score (Figure 3C) or Beta diversity (Figure 3D) between colic/crying groups.

Specific microbial taxa were differentially abundant and elevated in the colic and crying group, compared to the unaffected group, and were all aerobic, non-spore forming bacteria (Table 3). Three of these taxa were identified as phyla *Proteobacteria* class *Gammaproteobacteria* and were from the families *Burkholderiacceae* (*p* = 0.001), *Halomonadaceae* (*p* = 0.012), and *Moraxellaceae* (*p* = 0.022). The other more abundant taxa in the colic and crying group were from phyla *Bacteroidota* family *Weeksellaceae* (*p* = 0.015) and phyla *Firmicutes* family *Streptococcaceae* (*p* = 0.032). Two additional taxa were less abundant in the crying only group, compared to the unaffected group, and were identified as phyla *Actinobacteriota* families *Coriobacteriaceae* (*p* = 0.015) and *Mycobacteriaceae* (*p* = 0.045).

**Table 3:** Differentially abundant microbial taxa between colic/crying groups using the discrete false discovery rate (DS-FDR) method.

OTU Taxonomic Classification^a^	Metabolism	Spore Formation	Crying Only Mean rank difference^b^ (raw p)	Colic only Mean rank difference^b^ (raw p)	Colic and Crying Mean rank difference^b^ (raw p)
p__*Proteobacteria*; c__*Gammaproteobacteria*; o__*Burkholderiales*_592522; f__*Burkholderiaceae*_A_592522	Aerobic	Non-spore forming	0.00016 (*p* = 0.776)	0.00011 (*p* = 0.708)	0.00227 (*p* = 0.001)
p__*Proteobacteria*; c__*Gammaproteobacteria*; o__*Pseudomonadales*_641030; f__*Halomonadaceae*_641030	Aerobic	Non-spore forming	−0.00053 (*p* = 0.648)	−0.00063 (*p* = 0.386)	0.00521 (*p* = 0.012)
p__*Proteobacteria*; c__*Gammaproteobacteria*; o__*Pseudomonadales*_660879; f__*Moraxellaceae*; g__*Acinetobacter*	Aerobic	Non-spore forming	0.00237 (*p* = 0.749)	0.00039 (0.965)	0.02038 (*p* = 0.022)
p__*Bacteroidota*; c__*Bacteroidia*; o__*Flavobacteriales*_877923; f__*Weeksellaceae*; g__*Cloacibacterium*	Aerobic	Non-spore forming	0.00099 (*p* = 0.380)	−0.00095 (*p* = 0.209)	0.00802 (*p* = 0.015)
p__*Firmicutes_D*; c__*Bacilli*; o__*Lactobacillales*; f__*Streptococcaceae*; g__*Streptococcus*	Aerobic	Non-spore forming	0.00042 (*p* = 0.643)	−0.00010 (*p* = 0.878)	0.00246 (*p* = 0.032)
p__*Actinobacteriota*; c__*Coriobacteriia*; o__*Coriobacteriales*; f__*Coriobacteriaceae*; g__*Collinsella*	Anaerobic	Non-spore forming	−0.00080 (*p* = 0.015)	−0.00003 (*p* = 0.874)	−0.00003 (*p* = 1.000)
p__*Actinobacteriota*; c__*Actinomycetia*; o__*Mycobacteriales*; f__*Mycobacteriaceae*; g__*Corynebacterium*	Aerobic	Non-spore forming	−0.00639 (*p* = 0.045)	−0.00226 (*p* = 0.238)	0.00230 (*p* = 0.522)

^a^ Taxonomic classification was at either the genus or family level. Taxonomy is abbreviated as follows: p, phylum; c, class; o, order; f, family; g, genus.

^b^ Mean rank difference was calculated compared to the unaffected group. Values that have a *p* value <0.05 are colored red.

### 3.3 Additional cord blood biomarkers with no association with colic groups

We analyzed a wide range of additional biomarkers selected for their potential associations with colic, inflammatory pathways and/or neurologic pathways and found no association with the incidence of colic and/or excessive crying in the first six months of life (Table 2). These biomarkers included cord blood lymphocyte proliferation after allergen stimulation, cytokine production by cord blood lymphocytes after allergen stimulation, and cord blood metabolites. We also considered cord blood CRP in our analysis but did not have a sufficient sample size across the four colic and crying groups to assess this association.

## 4 Discussion

This exploratory study analyzed a range of immune- and neurologic-related biomarkers in cord blood to assess their potential involvement in the underlying mechanism(s) of infant colic. Our results suggest different fatty acid percentages and microbial signatures in cord blood. Infants with colic, as compared with excessive crying without colic and unaffected infants, had higher trans fatty acids compared with the unaffected group. We also detected differences in the microbial diversity and abundance measured in the cord blood samples, in that the colic-only group had the lowest microbial diversity compared to all other groups. The colic and crying group also had elevated abundance of *Gammaproteobacteria* compared to the unaffected group. We believe our results support an association of colic with both maternal dietary fatty acid consumption during pregnancy and distinct umbilical cord blood microbial signatures. Our findings in cord blood, an exposure which precedes the clinical manifestation of colic, also support our hypothesis that infant colic may be indicative of larger underlying mechanism(s). This is a theme we have explored in other analyses of postnatal outcomes (6, 14). Future work will be necessary to establish if infant colic is not merely a benign, self-resolving condition, but rather is an earlier indicator of a larger immune and/or neurologic dysbiosis.

Our findings suggest that differences in cord blood fatty acid percentages are most profound in infants exhibiting colic only, as compared with infants who are unaffected and those with excessive crying. Specifically, our data describe that higher percentages of trans fatty acids in cord blood are associated with the development of colic without excessive crying in infancy. To our knowledge, this is the first report of fatty acid levels specifically related to infant colic. Trans fatty acids, which are acquired exogenously through the maternal diet from a range of foods including hydrogenated oils, have also been shown to decrease the level of omega-3 long-chain polyunsaturated essential fatty acids (LC-PUFA) and are associated with poor health outcomes in infants as they develop (28). This is also consistent with our results which found increased trans fatty acids in the colic only group. It is important to note that trans fatty acids at the time of sample collection, in 1999–2002, were primarily acquired through dietary sources containing partially hydrogenated oils (PHO). However, the United States Food and Drug Administration has since identified the harmful impact of these artificial trans fatty acids (29). Since 2021, food manufacturers in the United States are no longer allowed to include PHOs in food production (29). Natural trans fatty acids can still be acquired through consumption of food products from ruminant animals, such as butter and cheese.

Considering that trans fatty acids decrease the level of LC-PUFA, it is also worth noting the prior evidence for underlying relationships between LC-PUFA consumption and infant behavior: Specifically, supplementation of DHA and AA, two LC-PUFA, during pregnancy or in infancy has been shown to reduce infants’ sleep disturbance score, suggesting that fatty acids play a role in infant comfort and soothability (30). In addition, mothers with higher LC-PUFA levels in their breastmilk had infants with less negative affectivity (31). In contrast, long chain monounsaturated fatty acids such as oleic acid (M6) in the adult colon is associated with intestinal pain (32, 33). Our study did not find an association between the colic and crying groups and the levels of DHA, AA or M6 in cord blood, which may be due to our sample timing at birth vs. earlier *in utero* or later during infant feeding, or due to the location of fatty acids in the intestines after feeding but not necessarily in circulation. All told, our findings in the context of others may support further research into the roles of different fatty acids in the underlying mechanism of colic.

Cord blood microbial signatures in the Viva cohort were also associated with colic groups, and may be related to some studies of probiotics for its treatment (34–36). Specifically, our data showed that lower diversity in cord blood microbial signatures, and differential abundance of specific microbial families, were associated with the development of colic without excessive crying in infancy. We measured microbial signatures in cord blood, which is distinct from the intestinal microbiome. However, prior studies have identified a role for the intestinal microbiome in colic through bacterial production of short chain fatty acids, which promote intestinal epithelial barrier integrity (37), dampen intestinal pain perception (38, 39) and block pathogen adhesion (40, 41). Probiotic strains of *Lactobacillus reuteri* (42) and *Bifidobacterium longum* (43) have successfully decreased infant crying duration and altered the intestinal microbiome. Another study supplemented breastfeeding mothers of colicky infants with a strain of *Actiregularis* in a single-blind randomized control study (44). In each of these studies, the frequency and duration of crying decreased and the infant bacterial diversity in stools increased. Our data in the context of these prior studies support a microbial influence at different sites on the development or prevention of colic. This mechanism of cord blood microbial signatures on the development or prevention of colic requires additional exploration.

In this study, we measured microbial signatures in cord blood, as opposed to in stool samples. Microbial signatures in cord blood and the intestinal microbiota are distinct sites, and we explored the possibility that there may be a microbial signature in cord blood that is relevant to colic. However, we state these findings with caution because the origin of the cord blood microbial signature cannot be determined from our study design. There are possible two sources of the cord blood microbial signature. It could indeed come from a microbiome present in the umbilical cord blood, which has been rarely studied (45, 46). A recent study identified microbial signatures in placenta samples from 54 pregnant women and found that the placental microbial signature was more similar to the vaginal microbiome in preterm births and more similar to the oral microbiome in term birth (47), supporting that this microbial signature is indeed indicative of a placental microbiome. Alternatively, the microbial signature measured could be from microbial DNA contamination acquired during the nonsterile cord blood collection process. Another recent study compared the microbial signatures measured across 15 different 16S rRNA sequencing datasets of placental samples and found a correlation between placental microbial signatures and the mode of delivery, strongly supporting contamination as the origin of these signatures (48). Either way, supporting the relevance of cord blood microbial signatures, two prior Project Viva studies have found associations of cord blood microbial signature diversity and species abundance with maternal atopy (20) and cord blood mononuclear cell IL-13 production (21). Regardless of the microbial source, our results here support a role for microbial function in the later development of colic.

In this study, we compared groups: colic with excessive crying, colic only, excessive crying only, and unaffected. In our previous studies in a larger sample of Project Viva participants, not restricted to availability of cord blood assay results, we combined both colic groups into one group, colic with or without excessive crying (14) because we found that maternal predictors of colic, such as maternal atopy (14), and clinical outcomes of colic, such as eczema, allergic rhinitis and asthma, are similar in the two colic subgroups (+/- excessive crying) (6). However, in our analysis of the different cord blood biomarkers, we identified different biomarker associations in these two colic subgroups. Specifically, we found that the colic only group had higher cord blood trans fatty acids and microbial signature diversity. These differences suggest that colic with and without excessive crying may have distinct underlying pathogenesis, which may be measurable in a biomarker but not distinguishable clinically. Further validation is necessary to determine whether these colic subgroups are biologically and/or clinically distinct.

We specifically explored biomarkers in cord blood as a timepoint before the onset of colic and crying symptoms. We have previously published results which showed several maternal and prenatal factors as strong predictors of colic, suggesting that there is a developmental pathway for colic beginning in gestation (14). Our hypothesis for this analysis was that colic is one indicator of later immune and/or neurologic dysregulation, and there are additional underlying mechanisms developing silently before and during these colic and crying signs. It is important to note that the majority of the candidate biomarkers we identified *a priori* were not associated with infant colic. These biomarkers included cord blood lymphocyte proliferation after allergen stimulation, cytokine production after allergen stimulation, and metabolites. All were collected in cord blood during delivery, and their lack of association with the colic groups may suggest that pathogenesis of colic at the time of birth does not include inflammatory processes or immune dysregulation. It is also worth noting that many of the sample sizes for these biomarkers were small and therefore the analyses had low power. Future studies involving biomarker measurement as colic and excessive crying behaviors manifest (e.g., around 3–6 months of age), and with larger sample sizes are needed to provide additional insights into underlying immune- or neurologic-mechanisms of colic.

This study had several limitations. First, sample sizes were small, especially in the colic subgroups. We suggest that future evaluations of cord blood fatty acids and microbial signatures in a larger sample size will be important to validate our findings. Many of our null findings, such as cytokine production after allergen stimulation, had very low power as a result. Second, we did not adjust significance levels for multiple testing because we examined correlated exposures, and because we were primarily interested in the strength, direction, and consistency of associations rather than examining *p* values. Third, our biomarker measurements were limited to a set number of biomarkers and only in cord blood, due to availability of already performed assays. We selected cord blood as the biological specimen due to its proximity to the onset of colic and crying symptoms, and the possibility for a predictive biomarker. Unfortunately, we did not collect infant blood in Project Viva. For this reason we also chose to use an unadjusted multinomial regression for our analysis due to the limited sample availability and exploratory nature of the study. Additional untargeted analysis of cord blood biomarkers, and measurement in blood collected at 3–6-month timepoints during the colic and crying symptoms will be beneficial for colic biomarker discovery. Fourth, cord blood collection is a non-sterile process. As a result, we cannot determine if the microbial DNA fingerprints associated with the colic groups indicate microbial exposure *in utero* or microbial exposure in the delivery room. Finally, the Project Viva cohort used a nonstandard definition of colic which limits our ability to compare results with those of other studies identifying colic using different criteria. However, as we have published previously, this definition, based on abdominal discomfort accompanying unsoothable crying, with or without excessive crying, is consistent with current models of disorders of gut-brain interaction and may be more accurate and descriptive (6, 14).

In conclusion, use of a longitudinal study of infants enrolled in a pre-birth cohort allowed us to explore the relationship between candidate predictive biomarkers in cord blood and colic and crying behaviors around 6 months of age. Our exploratory analysis identified higher trans fatty acid levels and *Gammaproteobacteria* microbial signatures in cord blood of colicky infants, and suggests these may be predictive biomarkers of colic. Importantly, we also found a lack of association of most of the immune and neurological biomarkers that we assessed in cord blood with infant colic. In turn, our results support fatty acid pathways and microbial diversity as underlying mechanisms in the development of colic in infants. Future work, such as analyzing the infant stool microbiome and blood fatty acid levels at 6 weeks of age, is necessary to validate these findings and continue an exploration for useful biomarkers of the common and distressing infant condition of colic.

## Data Availability

Publicly available datasets were analyzed in this study. This data can be found here: https://www.projectviva.org/for-investigators.
