# Supplementary material for: Associations of cord blood biomarkers with infant colic and excessive crying
Source: Front Pediatr. 2026 Apr 10;14:1767660. doi: 10.3389/fped.2026.1767660 (PMC13106567; doi:10.3389/fped.2026.1767660)
Supplement: Supplementary file 1 [file Datasheet1.pdf]

Supplementary Material

1 Supplementary Table

1.1 Supplementary Table 1: Biomarker sample sizes

Biomarker Assay (total n)	Unaffected n (%)	Excessive crying only n (%)	Colic only n (%)	Colic and crying n (%)
**Lymphocyte proliferation (n=282)**	183 (64.9%)	29 (10.3%)	45 (16.0%)	25 (8.9%)
**Cytokine production (n=225)**	147 (65.3%)	24 (10.7)	35 (15.6%)	19 (8.4%)
**Fatty Acids (n=261)**	170 (65.1%)	23 (8.8%)	43 (16.5%)	25 (9.6%)
**Metabolites (n=90)**	60 (66.7%)	11 (12.2%)	11 (12.2%)	8 (8.9%)
**16S Microbial Quantification (n=175)**	116 (66.3%)	17 (9.7%)	27 (15.4%)	15 (8.6%)
